# The Hidden Threat in Schools: Understanding and Managing Toxic Teacher Behaviors

**DOI:** 10.3390/bs15070838

**Published:** 2025-06-20

**Authors:** Osman Tayyar Çelik, Tamer Sarı, Seyfettin Abdurrezzak, Ümit Doğan, Ahmet Alper Karagözoğlu

**Affiliations:** 1 Department of Child Development, Faculty of Health Sciences, Inonu University, 44000 Malatya, Türkiye; 2 School of Foreign Languages, Pamukkale University, 20160 Denizli, Türkiye; tamersari@gmail.com; 3 Ministry of National Education, 06690 Ankara, Türkiye; srezzak@hotmail.com (S.A.); doganumit18@hotmail.com (Ü.D.); alperkaragozoglu@gmail.com (A.A.K.)

**Keywords:** toxic teacher behaviors, school principal, organizational toxicity, phenomenology

## Abstract

This study aimed to examine teachers’ toxic behaviors and their impact on the school environment from the perspective of school principals. The study, employing a phenomenological approach, was based on the views of 12 school principals in Türkiye. It was found that toxic behaviors were consistent with personality traits known as the dark triad—Machiavellianism, narcissism, and psychopathy—and were associated with characteristics such as low performance, professional inadequacy, and a lack of social skills within the school context. Findings suggested that such behaviors can have serious negative effects on organizational climate, teacher collaboration, and organizational trust. Particularly, according to the school principals’ views, toxic teachers poisoned the school environment, reduced motivation, and negatively affected the overall performance of the school. The study also evaluated the strategies school principals used to cope with toxic teachers, revealing a phased adoption of constructive and discipline-based intervention approaches. The results underscored the critical importance of school principals’ ability to effectively manage toxic behaviors for the academic and social success of their schools.

## 1. Introduction

Educational environments are dynamic systems in which the quality of human relationships significantly influences outcomes such as student achievement, teacher performance, and overall school climate. The role of school principals in maintaining a positive and functional school environment is crucial, especially when dealing with toxic behaviors from teachers. Toxic behaviors in the workplace have been extensively studied in the context of leadership and general organizational behavior, but there is a growing recognition that these behaviors can also emerge from non-leadership roles, such as teachers, with similarly destructive consequences. This study aims to explore the phenomenon of toxic teacher behaviors from the perspective of school principals, providing considerable insight into how such behaviors affect the educational environment and the strategies school principals use to mitigate them.

Workplace toxicity, a well-established concept in organizational psychology, has profound implications in educational settings, where it can disrupt teacher collaboration, student outcomes, and school climate (Reio & Reio, 2018; Mannix-McNamara et al., 2021). In Türkiye, where educational systems face challenges such as high teacher turnover and cultural expectations of teacher authority (Akiba et al., 2023), toxic behaviors among teachers exacerbate organizational dysfunction and undermine school effectiveness. Despite growing interest, research on toxic teacher behaviors remains limited, particularly in the Turkish context, highlighting the need for this study to explore these behaviors from the perspective of school principals. In this context, the answers to the following questions were sought:

How do school principals define toxic teacher behaviors and toxic teachers?

What are the effects of toxic teacher behaviors according to school principals?

How do school principals cope with toxic teacher behaviors?

## 2. Literature Review

### 2.1. Defining Toxic Behaviors

Toxic behaviors in the workplace are typically characterized by actions that harm the well-being, productivity, and morale of others. In the educational context, these behaviors can range from overt actions like bullying or gossiping to subtler forms of incivility, such as passive resistance or intentional underperformance. Research has identified several personality traits that are commonly associated with toxic behaviors, including the dark triad of Machiavellianism, narcissism, and psychopathy (Jonason et al., 2012). These traits are often manifested in manipulative, self-serving behaviors that prioritize personal gain over the well-being of the organization or its members.

In schools, toxic behaviors can have far-reaching effects, impacting teachers, students, and the broader educational environment. Research indicates that toxic teacher behaviors reduce collaboration among staff (Bahadır & Kahveci, 2020), diminish organizational trust (Mannix-McNamara et al., 2021), and increase teacher turnover (Sulea et al., 2012).

### 2.2. The Impact of Toxic Behaviors on School Culture

A significant body of research has explored the impact of toxic behaviors on organizational culture. In schools, where collaboration and teamwork are essential for success, toxic behaviors can undermine these efforts by creating divisions among staff and fostering an environment of mistrust. Toxic behaviors in schools can disrupt communication, escalate conflicts, and lower morale (Reio & Reio, 2018; Mannix-McNamara et al., 2021).

These negative outcomes not only affect the teachers and school principals but also have a trickle-down effect on students, who may become disengaged from their education because of the toxic environment.

Toxic behaviors can contribute to organizational deviance, where individuals act contrary to institutional norms and expectations (Demirdağ, 2018; Taştan, 2017). In schools, this might include teachers refusing to follow school policies, engaging in unprofessional behavior with colleagues, or neglecting their duties to students. Such deviant behaviors not only harm the immediate work environment but also erode the overall culture of the school, making it more difficult for school principals to foster a positive and productive learning environment.

While much of the literature on organizational behavior emphasizes the importance of positive dynamics in promoting well-being, it is equally critical to recognize that a sole focus on positivity may obscure underlying issues. Addressing only the positive aspects of behavior can inadvertently overlook the presence and impact of toxic actions, thus limiting our understanding of the broader organizational climate. This brings us to the discussion of positive psychology and its limitations in comprehensively dealing with workplace well-being.

The field of positive psychology focuses on the positive aspects and characteristics of people. This discipline aims to understand and promote positive experiences that support the development of individuals, organizations, and societies (Kobau et al., 2011). As in different disciplines, the organizational behavior literature naturally focuses on positive aspects such as workplace happiness, subjective well-being, and commitment. As a matter of fact, there has been a tremendous increase in research focusing on the positive emotions of education employees (Dilekçi & Manap, 2022; Hernández-Torrano, 2020). Along with this increasing interest, criticism of positive psychology has been increasing (Schyns, 2015). It is reported that positive psychology tends to ignore negative emotions and contextual factors, which may lead to an unwillingness to confront broader social problems (Lomas et al., 2020; Yakushko & Blodgett, 2021). In this context, as Wong (2019) emphasizes, we argue for the importance of addressing the darker aspects of human experiences to sustain the well-being of individuals and organizations. Focusing only on practices that promote positive emotions may not work. In addition, research has revealed that negative emotions and damaging behaviors can be a significant barrier to employee well-being (Celebi Cakiroglu & Tuncer Unver, 2024; Rasool et al., 2021). Therefore, focusing on negative emotions and behaviors may be a more integrative way to promote employee well-being. Recent studies have shown that members of organizations may exhibit intentional behaviors to harm colleagues and the organization (Özdemir & Demircioglu, 2015; Serenko & Abubakar, 2022). Toxic behaviors such as unethical behaviors, workplace incivility, revenge, jealousy, workplace aggression and bullying, and counterproductive work behaviors have become important problems that affect the productivity and functioning of today’s organizations. It may not be enough to focus on positive emotions to increase employee well-being; taking negative emotions and behaviors into consideration may offer a more holistic and effective approach.

### 2.3. Toxic Teacher Behaviors

Although toxic leadership has been extensively studied in relation to the dark triad (Milosevic et al., 2020; Snow et al., 2021), research on toxic teacher behaviors—though sparse—identifies behaviors such as gossiping, sabotaging colleagues, and resisting change as prevalent in schools (Mannix-McNamara et al., 2021; Sanyal, 2018). These behaviors, often linked to dark triad traits (Jonason et al., 2012), disrupt school culture by fostering conflict and reducing trust (Datta et al., 2017). For instance, Sanyal (2018) highlights how toxic teacher behaviors impede student development, while Datta et al. (2017) note their role in increasing teacher stress and student disengagement. However, as Appelbaum and Roy-Girard (2007) note, toxic behaviors are not limited to leaders; they can also emerge from lower-level employees, including teachers.

Toxic behaviors in educational and other organizational settings have predominantly been examined in the context of leadership and managerial behaviors (Green, 2014; Hattab et al., 2022; Milosevic et al., 2020; Snow et al., 2021). However, research specifically addressing the toxic behaviors of employees within these structures is relatively scarce. Jonason et al. (2012) explored the influence of tactics employed by toxic employees on business organizations, while Poursafar and Afkaneh (2020) investigated the typology of toxic employees in public organizations, concluding that personal, family, organizational, and environmental factors contribute to the formation of such behaviors. Similarly, Bayrakçı (2017) identified abusive behaviors as toxic in healthcare workers, and Arda and Kanten (2023) classified toxic behaviors based on personality traits, such as aggressive, depressive, hysterical, reactive, role model, ego-dominant, and ego-passive characteristics. Taştan (2017), in mixed-method research conducted in the health sector, defined toxic behaviors as actions including destroying others, destructive gossip, insidious policies, excessive negativity, abusive supervision, unfair policies, and aggression.

While these studies offer insights into toxic behaviors across various organizations, our understanding of toxic behaviors among teachers remains notably limited. Most of the existing research has focused on toxic leadership rather than examining the behaviors of educators themselves. This gap in the literature highlights the urgent need for more research on toxic teacher behaviors, particularly from the perspective of school principals who are often responsible for managing and mitigating these behaviors.

In one of the few studies to address this issue, Mannix-McNamara et al. (2021) found that toxic teacher behaviors, such as gossiping, sabotaging colleagues, and resisting change, were prevalent in many schools. These behaviors were often linked to personality traits associated with the dark triad, as well as other negative traits such as low emotional intelligence and a lack of empathy. The study also found that these behaviors had a significant impact on school culture, leading to increased conflict among staff, reduced trust in leadership, and a general decline in the quality of the educational environment.

### 2.4. School Principals’ Role in Managing Toxic Behaviors

Given the detrimental effects of toxic teacher behaviors on school culture, it is crucial to explore how school principals can effectively manage these behaviors. Research suggests that school principals often employ a variety of strategies to mitigate the impact of toxic behaviors, ranging from direct interventions, such as disciplinary actions, to more indirect approaches, such as fostering a positive school culture that discourages toxic behaviors (Izadi & Hart, 2023).

School principals employ diverse strategies to manage toxic teacher behaviors, ranging from open communication to disciplinary actions (Bozkurt et al., 2020; Powers, 2000). Unlike Bozkurt et al.’s (2020) focus on organizational trust as a preventive measure, this study examines how principals in Türkiye adopt a phased approach, combining constructive and disciplinary interventions, to address toxic behaviors. Similarly, Powers (2000) advocates for clear behavioral expectations, aligning with the formal policies observed in this study’s findings, although contextual differences in Türkiye’s educational system shape unique implementation challenges.

### 2.5. Cultural and Contextual Considerations

One aspect that the literature on toxic behaviors often overlooks is the role of cultural and contextual factors in shaping how these behaviors are perceived and addressed. In some cultures, for example, toxic behaviors may be more readily tolerated or even normalized, while in others, they may be seen as completely unacceptable (Sanyal, 2018). This is particularly relevant in the context of education, where cultural attitudes toward teachers can vary significantly.

In cultures where teachers are held in high esteem, such as in many Asian countries, toxic behaviors from teachers can have a profound impact on students, potentially undermining their respect for authority and disrupting their learning (Reio & Sanders-Reio, 2011). In contrast, in cultures where teachers are not as highly valued, such as in some parts of the United States (Akiba et al., 2023), the impact of toxic behaviors may be less pronounced, as students and parents may not place as much importance on the teacher’s behavior.

This highlights the need for more research on how cultural and contextual factors influence the prevalence and impact of toxic teacher behaviors. By understanding these factors, school principals can develop more effective strategies for managing toxic behaviors that are tailored to the specific needs of their schools and communities.

School principals, while ensuring school effectiveness, not only focus on success but also try to address teachers’ interests and needs and organizational issues, and work on shaping the school culture, changing teacher attitudes, and reducing destructive behaviors. However, to eliminate, reduce, or prevent toxic behaviors, it is important to understand these behaviors and structures and their potential effects (Appelbaum & Roy-Girard, 2007). This study is significant in that it is the first to focus on teachers’ toxic behaviors from the perspective of school principals. In addition, identifying toxic teacher behaviors can contribute to understanding toxic behaviors in the school environment, developing intervention strategies to minimize such behaviors that reduce teacher performance, and creating a positive school climate. This study did not adopt a single lens of toxic organizational culture, toxic personality traits, or toxic leadership. Instead, these structures were recognized as conditions that pave the way for toxic behaviors, and toxic behaviors in the school context were defined as those that directly harm individuals, reduce job performance, and affect relationships among families, students, and colleagues, and the achievement of school goals.

## 3. Materials and Methods

Phenomenological research aims to understand people’s experiences by accessing their lifeworlds (Christensen et al., 2014; Creswell, 2017a). This study employed a phenomenological research design since it aimed to understand toxic teacher behaviors from the perspective of school principals and how these behaviors affect the institution, coworkers, and other stakeholders. A significant feature of phenomenological studies is their goal of finding the common aspects and essence of shared experiences. Phenomenology assumes that people interpret similar experiences in a similar way; hence, it aims to find the commonality in these experiences (Fraenkel et al., 2012). In this study, the phenomenon of interest was the toxic behaviors of teachers. The shared experiences were the toxic teacher behaviors experienced by school principals at different school levels. The study aimed to involve the reader in the process of in-depth description of school principals’ experiences with toxic teachers. The requisite permission from the ethics committee for the study was obtained.

### 3.1. Participants

The participants comprised 12 school principals working at different school levels in Turkey in the 2022–2023 academic year. While determining the participants of the study, the selection criteria for phenomenological studies proposed by Moustakas (1994) were taken into consideration. These criteria are: (1) the participants have intensive experience with the phenomenon, (2) they are interested in understanding the phenomenon, and (3) they are willing to participate in the study and publish the findings. In this context, the snowball sampling method was used to identify school principals (Creswell, 2017b). The selection was strategic as it was based on the recommendation of a supervisor of the supervisory board of the provincial directorate of national education. Supervisors are experienced in getting to know schools as they handle complaints and investigations and are involved in school counselling services. The first participant was a school principal who had dealt informally and formally with toxic individuals in their school. This strategy was also used for subsequent participants. Wilson (2015) states that there is no definite rule for the number of participants in phenomenological research; this number can be between 6 and 20 people. In addition, data saturation can be taken as a criterion for terminating the data collection process (Yıldırım & Şimşek, 2016). Data saturation is the absence of new codes and themes that serve the purpose of the research due to the repetition of similar opinions (Dömbekci & Erişen, 2022). In this context, data collection was limited to 12 participants. Information about the participants is shown in Table 1.

### 3.2. Data Collection

Data were collected using a semi-structured interview form. Interviews are a data collection method that is conducted with individuals face-to-face or via technological/digital tools such as telephone, etc., and enable the collection of in-depth information on a specific topic (Kvale & Brinkmann, 2009). Participants’ views were elicited through open-ended questions developed by the researchers, which allowed them to express themselves without being limited by the researcher’s perspective or findings from previous research (Creswell, 2017b). A semi-structured interview format was preferred as it allowed the participants’ unique perspectives to be explored and provided the opportunity to ask sub-questions during the research.

The first draft of the interview form consisted of eight questions and was presented to two associate professors of educational administration and one professor of educational psychology for expert feedback. The form was revised according to experts’ suggestions, and the number of questions was reduced to six. After these revisions, the form was finalized after a pilot study with a school principal. Example interview questions in the form are as follows:

1. How would you define toxic teacher behaviors?
2. Have you experienced such toxic behaviors in your school? If so, could you share these experiences?
3. How do the toxic behaviors you have observed/experienced in teachers in your school affect the school environment?
4. What kind of approaches do you take in the face of toxic behaviors exhibited by teachers?

Before the interviews, the participants were called, and the purpose of the study was explained. It was stated that participation was voluntary and that personal information would be kept confidential. The date, time, and place of the interview were determined according to the preferences of the participants. Before the interviews, a “Participant Interview Consent Form” was presented to the participants along with the interview questions, and this form was signed by the researcher and delivered to the participants. This document ensures the confidentiality of the participants and guarantees their right to terminate the interview at any time.

The interviews were conducted by two researchers in school and private settings during the spring semester of 2022–2023. The interviews were conducted in line with the interview guide and lasted between 30 and 47 minutes. To ensure participants could express themselves comfortably, we implemented several measures. These included providing assurance regarding participant confidentiality and emphasizing the non-judgmental nature of the research and interviews. Interviews were recorded with participant consent, and notes were simultaneously taken by a researcher. Additionally, active listening techniques were employed to examine and understand participants’ experiences in depth, and clarifying questions that were not part of the interview protocol were posed to participants. Audio recordings were manually transcribed verbatim by researcher AK. Manual transcription enabled the capture of emotions, contextual details, and nuances present in the interviews. Subsequently, transcripts were emailed to participants for verification. While nine participants made no modifications, three participants provided additional clarifications via email. Each participant was given a pseudonym to protect their privacy.

### 3.3. Data Analysis

Analysis began immediately after each interview transcript was sent to the respective school principal for approval, without waiting for other interview transcripts to be completed. The data analysis followed a three-stage process proposed by Miles et al. (2019): data condensation, data display, and inference and validation. The data condensation phase involved simplifying, summarizing, coding, and categorizing the data. In this phase, each researcher read the interview notes and transcripts repeatedly to familiarize themselves with the data. Thematic analysis was used to code the data. Thematic analysis involves coding the data under predefined themes or themes developed during the data analysis process (Yıldırım & Şimşek, 2016). Three themes were identified in line with the research questions: characteristics of toxic teachers, effects of toxic teachers, and coping strategies. All researchers participated in the coding process. Initial coding was performed using the “in vivo” coding method, where participants’ own words or short statements were used as codes (Miles et al., 2019). For example, codes expressing the effects of toxic teacher behaviors include “...other teachers hesitate to speak in the presence of such teachers...”, “...these teachers undermine the school atmosphere...”, and “...other teachers avoid collaborating with them on joint projects...” In the subsequent phase, these brief codes were renamed by connecting them to existing concepts in the literature. For instance, the codes mentioned above were recoded as “organizational trust”, “organizational climate”, and “teacher collaboration”. In the next coding cycle, new codes related to the themes were sought, and existing codes were recoded for conceptual fit. In addition, a different strategy was followed while coding the theme of school principals’ coping strategies with toxic behaviors. We observed that school principals applied different strategies gradually. Therefore, we coded which strategy they used at which stage separately. A list of themes and codes was created, and each researcher assessed the appropriateness of the codes under each theme. The codes derived from the statements directly expressed by the participants and the thematic codes, which were interpreted and renamed by the researchers in light of the literature, are presented in tables. The codes mentioned in the statements of the participants are indicated for the relevant participants in the table.

Reliability and validity are two fundamental elements of all research. In qualitative research, these two concepts are also evaluated within the framework of rigor or trustworthiness (Cypress, 2017; Golafshani, 2003). Lincoln and Guba (1985) point to four general criteria in approaches to trustworthiness and recommend the use of at least one strategy in accordance with these general criteria. These general criteria are credibility, transferability, dependability, and confirmability. Within the scope of credibility, researcher triangulation was made in this study as suggested by Riege (2003). Each stage of the research was rigorously conducted and evaluated by all researchers. In addition, for member checking, the report containing the research findings and interpretations was presented to the participants to evaluate its appropriateness. Participants provided positive feedback. Transferability is similar to external validity in quantitative research (Riege, 2003). Within the scope of transferability, the data analysis stages were explained in detail, and how themes and codes were created was reported. Dependability was ensured by presenting the report detailing all stages and results of the research process to three experts for peer review, as suggested by Cypress (2017). Confirmability is the verifiability of the data collection and analysis process and data-based inferences. In this context, the data collection and data analysis process were reported in detail. In addition, to prove confirmability, coding reliability was calculated using the formula suggested by Miles and Huberman [(Reliability Coefficient = Number of themes or terms agreed/(Number of themes or terms agreed + Disagreement) × 100]. According to the formula, the reliability of 52 codes was calculated as 90.38% [(90.38 = 48/52) × 100]. While the findings related to the themes of toxic teachers’ characteristics and toxic teachers’ effects were presented in tables formatted as matrices, the theme of coping strategies was visualized using a pattern table.

## 4. Results

### 4.1. Characteristics of Toxic Teachers According to School Principals

The first aim of our research was to define toxic teacher behaviors from the perspective of school principals. In this context, toxic teacher characteristics according to school principals were presented under this theme. All participants shared that they had experienced working with one or more teachers who exhibited toxic behaviors at various stages of their professional lives. The most frequently highlighted characteristics of teachers who exhibit toxic behaviors included manipulation, resistance to change, attempts to dominate, self-interest, spreading gossip, aggression, and destructive criticism. The common view among participants was that teachers with toxic personality traits typically hinder collaboration and a positive work environment. Participants mentioned that teachers with toxic personality traits displayed minimal performance while carrying out their duties, doing just enough not to get into trouble with laws, regulations, and statutes. Table 2 shows the personality traits and behaviors of toxic teachers according to the participants’ views.

The participants expressed that the most dangerous trait found in toxic teachers was their constant monitoring of school administration and teachers. They mentioned that this allowed them to gather data to defend themselves, prevent investigations against them, and stop colleagues from taking negative stances against them.

*A school principal expressed his/her experience regarding this matter as follows: “During a faculty meeting while discussing duty schedule matters, a teacher exhibiting toxic behaviors attempted to create pressure in the environment by saying things like “you said this,” “you implemented such a practice before,” and “you made this kind of decision about another colleague’s duties.”*

(Turgut)

From the perspectives of the participants, it was understood that professionally incompetent teachers who exhibit toxic behavior generally victimize the school administration, whereas those who were professionally competent tended to show their darker sides to department colleagues and other peers. The views of Participant 3 regarding teachers with toxic characteristics are as follows:

*“I have encountered teachers who constantly try to impose their own desires. If these are not embraced or accepted, they create unrest within the school, continuously criticize the school, the school principals, or other teachers, demean them, and even try to pit some parents against the teachers.”*

(Selim)

As seen in the participant’s statement, teachers exhibiting toxic behavior aim to dominate the school management and have a say in decisions through threatening behaviors, destructive criticisms, and demeaning attitudes. In addition, participants mentioned that some teachers remained neutral to protect themselves from the pressure of teachers exhibiting toxic behavior, while others tried to join the toxic group, believing it to be in their best interest. Another participant described constantly complaining and dissatisfied teachers exhibiting toxic behavior as follows:

*“They are dissatisfied. It shouldn’t be like this… They affect you like pressurized water, like a current. They are always complaining. They find nothing effective.”*

(Çiçek)

Another participant emphasized toxic teacher characteristics, such as using power centers for their interests, selfish behaviors, prejudice, gossip, and resistance to change, as follows:

*“They work for their personal interests by using points of power and influence in education (unions, politics, relatives...), and with this power, they attempt to manipulate others.”*

(Hakan)

Based on the participating school principal’s observations, it is evident that teachers exhibiting toxic behaviors can manipulate by leveraging various power centers, such as unions and politicians. Consequently, they serve their personal interests and objectives rather than the school’s goals. The following participant, however, emphasized toxic teachers’ focus on personal interests rather than collective objectives, highlighting this aspect over manipulative behaviors.

*“One of the most striking traits of these individuals is their selfish personality. They never care about the general goals of the institution; their personal comfort is always the priority, and they constantly come to us, the school principals, with demands that prioritize their personal comfort.”*

(Murat)

*“I think these teachers particularly develop a bias against school principals. They think that the school principal is bad anyway, I need to be on guard, I need to criticize what they say. I believe they reflect their past problems, familial issues, and various daily problems they encounter onto the school. These types of teachers can be completely closed to new ideas, they criticize what you do without seeing the results, without consideration, and these criticisms generally occur not face-to-face but as gossip.”*

(Deniz)

From the above quotation, it is understood that teachers who exhibit toxic behaviors tend to criticize and judge school principals in particular. In addition, other points emphasized by the school principals were that toxic teachers may be closed to innovation and may harm the healthy communication environment of the school.

### 4.2. The Detriments of Toxic Teacher Behaviors

The second research question in our study pertained to the detrimental effects of toxic teacher behaviors. Findings derived from the participants’ views on the troubles caused by toxic teacher behaviors are presented in Table 3. These troubles included the respectability of the profession, institutional image, organizational climate, democratic environment, organizational culture, teacher turnover, organizational performance, organizational trust, teacher anxiety, organizational deviance, motivation, student transfers, parent-school cooperation, school principal authority, teacher collaboration, and creating an attractive environment. Accordingly, toxic teachers had multidimensional negative effects in educational settings. The participants expressed that teachers with toxic characteristics lacked the intention to achieve institutional goals and were indifferent to the general institutional objectives. In addition, they had a tendency to hinder collaboration and the creation of a positive working environment. They disregarded organizational culture and rules as values. Some of them intentionally neglected their duties or claimed to have forgotten them. Participants believed that toxic teachers’ gossip with external stakeholders damaged the institution’s privacy and image. Behaviors that narrowed the living space of non-supportive teachers, made them feel unsafe, and damaged their professional respectability were reported. The decrease in teachers’ energy and motivation was a consequence of the toxic environment. These individuals were identified as a significant cause of teacher turnover in schools. The participant school principals considered this situation a threat to school success.

Participant 7 described the detrimental effects inflicted by toxic teacher behaviors on the school climate and internal cooperation as follows:

*“They negatively affect the school climate with constant criticism and complaints, lowering employees’ motivation. By weakening the spirit of collaboration and solidarity, they foster factionalism. As a result, the institutional structure weakens.”*

(Hakan)

From this participant’s views, it is understood that teachers with toxic characteristics facilitate factionalism while using constant criticism and complaints as a tactic. In addition, Participant 11 emphasized that the gossip spread by toxic teachers was a leading behavior damaging the school climate and internal cooperation.

Another participant described the troubles caused by toxic teachers as follows:

*“The continuous violation of privacy in the school makes teachers feel unsafe. By creating a difficult work environment, it can stress the teachers and lead to psychological issues for some. They are preoccupied with the negatives in the school, hence do not spend sufficient time and effort on the students. They tend to neglect their educational duties.”*

(Remzi)

The participant particularly emphasized that teachers with toxic characteristics did not allocate time for students and neglected educational activities because they were focused on creating negativity and concentrating on negative situations. In addition, it was understood that the anxiety and stress created by toxic teachers among colleagues also led to teacher turnover.

The views of another participant on how toxic teacher behaviors damage parent-school cooperation are as follows:

*“The problems caused by toxic teachers lead to dissatisfaction among parents. They misinform students and parents by deliberately distorting events, which can negatively affect parent–school relations.”*

(Ahmet)

As can be understood from this, teachers exhibiting toxic behavior caused parents and students to take a stand against the school by misleading them with incorrect information. This also undermines parent–school cooperation. Participant 8 described the damage that toxic teachers did to the trust environment in the school as follows:

*“Toxic teachers can disrupt the administration–teacher relationships. Mistrust and disagreements negatively affect school management and make it difficult to achieve institutional goals.”*

(Murat)

### 4.3. School Principals’ Coping Methods with Toxic Teacher Behaviors

The third question of our research was about school principals’ methods of coping with toxic behaviors. Participants indicated that they attempted to cope with toxic teacher behaviors by ignoring them, compromising, giving verbal warnings, and taking official actions (such as initiating investigations). Some participants mentioned that teachers exhibiting toxic behavior perceive others as toxic and project these feelings onto others. It was understood that school principals have unique and step-by-step methods for dealing with toxic teacher behaviors. Since some toxic behaviors fall within legal norms, ignoring them and open communication were the first options school principals used for resolution. To prevent harm to the organization, school principals employed empathy, goodwill, sacrifice, initiative, and patience. Participants expressed that when a threat was felt on behalf of the institution, they requested investigations for offenses such as neglect of duty and mobbing. Table 4 presents the methods school principals use to deal with toxic teachers.

Participant 6 shared the method they used to deal with toxic teacher behaviors as follows:

*“I believe a good school principal should know the personal issues of all teachers. When a teacher comes to school, they cannot leave all their emotions and experiences outside. After all, we are human. I think school principals need to be more understanding. It’s crucial to build positive relationships with these individuals. Therefore, I get to know them closely and assign tasks that suit them. Empathy is necessary to gain their trust. I try to help them confront their behavioral issues. Later, I informally suggest psychological guidance.”*

(Deniz)

The statement indicates that Deniz adopted a humane approach. He believed that events not going well in life can reflect on the school. He gave toxic individuals tasks they could manage, followed a step-by-step process of getting to know them better, and provided guidance if needed. He did not resort to more rigid methods.

Participant 5, who emphasized a transparent management approach and open communication, shared their views as follows:

*“Effective communication between students, teachers, and school principals is of great importance. Problems should be solved through open and honest discussions. Teachers, students, and school principals trying to understand each other contribute to creating a positive school culture.”*

(Çiçek)

Here, Çiçek highlighted the importance of a transparent management approach and open communication to create a positive school culture. She believed that such a school culture would reduce toxic behaviors. It is understood that in line with this management approach, she tended to tolerate such behaviors and believe that toxic behaviors would eventually disappear within the school culture.

Another participant described how they try to cope with toxic teacher behaviors as follows:

*“I try to involve these individuals in various projects and tasks. At a more advanced level, I attempt one-on-one meetings. It’s necessary to remind them of their main duties, to tell them they are teachers and should focus on their students, and to show them the potential negative consequences when they interfere with others’ work, just as others would interfere with their work.”*

(Erdem)

Evidently, Erdem first applied the method of involving toxic teachers in work. In subsequent stages, he preferred direct sharing about what should be done and ultimately turned to legal measures, such as initiating investigations, when these methods were ineffective

## 5. Discussion

This study examined toxic behaviors exhibited by teachers from the perspective of school principals. Understanding the general characteristics of toxic teachers, their impact on the school and their colleagues, and how school principals cope with such individuals contributes to a deeper understanding of the multifaceted effects of toxic behaviors in educational settings. The findings provide a framework based on school principals’ views regarding toxic teacher behaviors, the impacts of these behaviors, and the coping methods employed by school principals.

The findings indicated that the characteristics of toxic teachers encompassed a wide range of behaviors associated with various personality structures and psychological traits. Behaviors such as manipulation, self-interest, destructive criticism, selfishness, and attempts to dominate, identified by school principals as toxic, align with the narcissistic and Machiavellian personality traits noted in the literature (O’Boyle et al., 2012; O’Reilly et al., 2014). Furthermore, toxic teachers exhibited aggression, hostile behaviors, psychological harassment, and threatening actions, which are associated with psychopathy, one of the dark triad traits (Mathieu et al., 2020; O’Boyle et al., 2012). In addition, principals’ definitions of toxic teacher behaviors overlap with the views of researchers who directly define toxic personality and its characteristics. For example, Rambuyon and Domondon (2021) define toxic personalities as those who exhibit ineffective work behaviors that harm the organization, teams, and individuals, while Arda and Kanten (2023) define toxic personality within the framework of perception management and influence tactics. As a result, this study reveals toxic teacher behaviors from the perspective of school principals in a manner consistent with different approaches to toxic behaviors.

The participants highlighted additional characteristics not heavily emphasized in the existing literature, such as resistance to change, prejudice, unwillingness to communicate, and inadequacy in professional and social relationships. These findings suggest that the contextual factors of the school environment may influence which traits are perceived as toxic. Contextual factors may include the expectations of school principals, shared values, and beliefs that express the school culture. Organizational cultures can become toxic when they develop destructive interactions, dysfunctional values and beliefs, and damaging traditions (Mannix-McNamara et al., 2021). In addition, teachers are a key element in achieving the school’s goals. Therefore, principals may identify teacher behaviors and characteristics that could undermine the achievement of goals as toxic. Similarly, in a study on the toxic leadership behaviors of school principals from the perspective of teachers (Alanezi, 2022), human relations skills, management skills, professional ethics, and authoritarian leadership were identified as the four main categories of toxic leadership. These results underline that the nature of the organization, its goals, and contextual conditions may be decisive in determining which behaviors are considered toxic. Finally, we stress the importance of addressing toxic teacher behaviors given that teachers’ professional efficacy plays a critical role in achieving a school’s goals (Sammons & Bakkum, 2011) and toxic behaviors can have far-reaching effects on organizational health (Brett & Stroh, 2003).

The findings of the study revealed that toxic teacher behaviors have a wide range of effects that can damage institutional activities and individual relationships. According to school principals, toxic teachers typically hinder collaboration and the creation of a positive working environment, damaging the image, organizational climate, culture, and trust in the organization. These results may be explanatory for previous studies (Padilla et al., 2007; Schmidt, 2014), which found that toxic behaviors increase job stress and decrease job satisfaction, organizational trust, and commitment. Indeed, a toxic school culture is characterized by poor communication and trust, conflict and hostility, fear and judgment (Yee & Yee, 2024). In addition, our results revealed that, according to school principals, toxic teacher behaviors may cause anxiety and turnover among teachers. Kaplan and Uğurlu (2022) found that a toxic school environment was associated with teachers’ lower life satisfaction. Therefore, the spread of toxic teacher behaviors may erode teachers’ motivation. In addition, the harmful effects caused by toxic individuals may create insurmountable difficulties over time, which may result in organizational cynicism that negatively affects communication, trust, and goal achievement (Özkaya & Kazak, 2023). Moreover, a toxic school culture may lead to decreased productivity and increased teacher turnover (Yee & Yee, 2024). Finally, our findings showed that toxic teacher behaviors may also cause students to change schools. Previous studies (Datta et al., 2017; Liaquat et al., 2024) also provide evidence that toxic teacher behaviors can affect students’ engagement in school activities, psychosocial well-being, and academic progress. As a result, these findings suggest that toxic teacher behaviors have multidimensional negative effects ranging from the school’s organizational structure to students’ engagement in school and can seriously threaten the overall functioning of the school and the formation of a healthy learning environment.

The third set of findings in the study focused on the methods used by school principals to deal with toxic teacher behaviors. The results revealed that school principals gradually adopted two main strategies: constructive methods and discipline-based methods. Constructive methods include positive approaches such as transparent management, involving teachers in decision-making processes, and open communication. When these methods prove ineffective, school principals resort to discipline-based approaches, such as warnings and disciplinary investigations. These strategies vary according to the context and situation, reflecting the individual approaches of principals.

Toxic behaviors encompass unethical attitudes, workplace incivility, and bullying, necessitating preventive approaches to combat these behaviors (Himmetoğlu & Bayrak, 2017; Serenko & Abubakar, 2022). Developing a collaborative school climate, supporting perceptions of justice, and detecting toxic behaviors at an early stage are crucial management strategies. Organizational transparency, practices encouraging participation, and positive approaches (Field, 2014; Gilbert et al., 2012) are effective tools in managing toxic behaviors. While these strategies generally encompass organizational-level measures, acknowledging individual needs can yield more effective outcomes. Listening to and actively communicating with toxic individuals fosters the development of personalized approaches (Powers, 2000).

According to Pearson et al. (2000), effective strategies for incivility in schools include preventive, interventionist, and supportive measures, with practices such as setting expectations and implementing zero-tolerance policies. School principals implement a phased approach, starting with preventive measures and moving towards interventionist strategies, to manage toxic behaviors. According to Saiti (2015), this process highlights the importance of using collaborative and adaptive methods in conflict resolution to improve overall school performance.

## 6. Conclusions, Implications, and Limitations

Toxic behaviors can create ripple effects within school organizations, potentially leading to the development of a harmful environment. This study aims to identify toxic teacher behaviors, their impacts, and the intervention strategies employed to address them from the perspective of school principals.. The findings indicated that behaviors and personality traits identified in the literature as toxic were also present in teachers. In addition, characteristics that are not emphasized much in the literature, such as professional and social incompetence and unwillingness to cooperate, were also considered toxic by school principals. The results revealed that toxic behaviors can have many negative effects at both the individual and organizational levels. According to the school principals, toxic behaviors can lead to a wide range of important consequences, starting from damage to the school’s institutional image to a decrease in teacher cooperation and motivation, to increased anxiety and weakening of organizational trust in teachers, and finally to the deterioration of parent–school cooperation. The study also showed that principals used a variety of methods to address toxic teacher behaviors, ranging from constructive interventions to discipline-oriented interventions, primarily based on their professional judgements and personal experiences. Although principals occasionally resorted to formal disciplinary processes, the most frequently used and initially favored strategies were individualized approaches developed in their own school contexts, such as open communication, strengthening relationships, and informal warnings.

Despite some limitations, this study provides important information for policy, practice, and further research. First, addressing toxic behavior in schools begins with recognizing such behavior, analyzing its causes, and selecting appropriate intervention methods. While the analyses in this study provide information about the elements that constitute toxic teacher behavior, contextual factors can determine what is considered toxic behavior, so this is not a universally applicable catalogue. Therefore, school principals must be careful observers and evaluators to correctly identify toxic behavior. Furthermore, our study implies that the spread of toxic behavior in the school environment can lead to a toxic school climate and culture. Dealing with a toxic school culture requires inspirational and transformative leadership strategies (Mette, 2020). In this context (primary school, secondary school, high school, and higher education), school leaders need to develop ethical principles, policies, and practices to create a safe and healthy school environment. In addition, different levels of education may have different teacher profiles and institutional cultures. Therefore, school leaders should consider contextual factors when developing strategies to address toxic behavior. Finally, policymakers can focus on developing in-service training programs to assist school principals in addressing and eliminating toxic behavior.

This study has some limitations. First, although our results provide a broad framework for the potential effects of toxic behavior, more longitudinal studies are needed to understand these effects in schools. Furthermore, while this study summarizes the intervention methods used by school principals, the effectiveness of these interventions in eliminating toxic behavior has not been determined. Future research could evaluate the effectiveness of intervention methods aimed at addressing toxic behavior and focus on the relationship between school administrators’ leadership styles and toxic teacher behavior. Second, this study was conducted in a context dominated by a centralized and hierarchical education system. Research on toxic teacher behavior and its effects in different cultural contexts and education systems could contribute to a better understanding of toxic behavior. Additionally, only one female school principal was included in the study, which may limit the diversity of experiences reflected in the findings. Future research could compare the experiences of male and female school principals.

In conclusion, this study fills an important gap in the literature by providing important insights into the effects of toxic teacher behaviors on school environments and how principals cope with such challenges. By examining toxic behaviors from the perspective of school principals, the results reveal the multidimensional negative effects of these behaviors on organizational climate, teacher well-being, and the overall functioning of the school. The results highlighted the critical role of principals by describing their methods of dealing with toxic teacher behaviors. Given that principals act largely autonomously in this process, the development of more structured, effective intervention policies that are aligned with institutional goals is a critical factor in combating toxic behaviors. To create healthy, supportive, and high-performing school environments, it is of great importance to address toxic behaviors comprehensively.

## Figures and Tables

**Table 1:** Demographic information of the participants.

Participant Number	Code Names	Gender	Education Status	School	Age	Year as School Principal
Participant 1	Mehmet	M	Master of Arts	Primary School	56	14
Participant 2	Ahmet	M	Master of Arts	Secondary School	50	21
Participant 3	Selim	M	Master of Arts	High School	45	17
Participant 4	Turgut	M	Master of Arts	Secondary School	52	17
Participant 5	Çiçek	F	Master of Arts	High School	52	17
Participant 6	Deniz	M	Master of Arts	High School	50	12
Participant 7	Hakan	M	Bachelor’s Degree	Vocational and Technical High School	55	6
Participant 8	Murat	M	PhD	Primary School	45	23
Participant 9	Remzi	M	Master of Arts	Primary School	45	14
Participant 10	Cihangir	M	Bachelor’s Degree	Vocational and Technical High School	61	27
Participant 11	Kenan	M	Master of Arts	Primary School	38	17
Participant 12	Erdem	M	Master of Arts	Secondary School	46	17

**Table 2:** The characteristics of toxic teachers according to school principals.

	P1	P2	P3	P4	P5	P6	P7	P8	P9	P10	P11	P12	*f*
Avoiding collaboration		✓	✓	✓	✓	✓	✓	✓	✓		✓	✓	10
Attempting to dominate	✓	✓	✓	✓	✓	✓	✓	✓		✓	✓		10
Spreading gossip	✓		✓		✓	✓	✓				✓	✓	7
Aggression	✓			✓	✓			✓	✓	✓	✓		7
Manipulation	✓	✓			✓				✓		✓	✓	6
Self-interest	✓			✓	✓		✓				✓	✓	6
Resistance to change			✓	✓	✓	✓			✓		✓		6
Being uncommunicative				✓		✓		✓		✓	✓	✓	6
Lack of productivity	✓						✓	✓		✓		✓	5
Accusatory behavior				✓	✓			✓			✓	✓	5
Destructive criticism					✓	✓				✓	✓	✓	5
Selfishness					✓			✓		✓	✓		4
Mocking and demeaning attitude	✓			✓							✓	✓	4
Threatening behavior				✓	✓			✓	✓				4
Displaying minimal performance				✓		✓	✓	✓					4
Dissatisfaction				✓		✓	✓	✓					4
Obstructing collaboration	✓	✓										✓	3
Discriminatory cliquing	✓					✓			✓				3
Neglecting duties		✓			✓		✓						3
Constant monitoring				✓				✓			✓		3
Psychological harassment				✓				✓			✓		3
Prejudiced attitude				✓						✓		✓	3
Touchiness				✓				✓					2
Jealousy							✓					✓	2
Professional incompetence							✓	✓					2
Spitefulness					✓								1
Violating norms				✓									1
Making excuses							✓						1
Inadequacy in social relationships										✓			1

**Table 3:** Effects of toxic teacher behaviors from the perspective of school principals.

	P1	P2	P3	P4	P5	P6	P7	P8	P9	P10	P11	P12	*f*
Institutional image		✓	✓	✓	✓	✓	✓	✓	✓	✓			9
Organizational performance		✓	✓	✓	✓	✓			✓		✓		7
Parent-school cooperation		✓		✓		✓		✓	✓	✓	✓		7
Organizational climate		✓					✓	✓	✓		✓	✓	6
Organizational trust				✓		✓	✓	✓	✓		✓		6
Teacher anxiety					✓	✓		✓	✓	✓			5
Motivation				✓	✓			✓			✓	✓	5
Student transfers			✓					✓		✓	✓		4
Teacher collaboration									✓	✓	✓	✓	4
Democratic environment				✓					✓	✓			3
School principals’ authority	✓							✓			✓		3
Creating an attractive environment						✓			✓		✓		3
Professional respectability	✓		✓										2
Organizational culture				✓				✓					2
Organizational deviance								✓			✓		2
Teacher turnover											✓		1

**Table 4:** School principals’ ways of dealing with toxic teachers.

	1. Step	2. Step	3. Step	4. Step
P1	Emphasis on the profession’s and institution’s prestige	Open communication	Initiating an investigation	
P2	Collaboration	Reminder of the rules	Initiating an investigation	
P3	Considering their requests	Tolerance and patience	Initiating an investigation	
P4	Ensuring participation in decisions	Transparent management	Tolerance	
P5	Ignoring	Patience	Psychological support and guidance	
P6	Transparent management	Open communication	Meeting their needs	
P7	Assigning tasks tailored to the individual	Understanding problems in their personal life	Psychological support and guidance	
P8	Ignoring	Open communication	Verbal warning	Initiating an investigation
P9	Collaboration and guidance	Verbal Warning	Open communication	
P10	Including them in social activities		Initiating an investigation	
P11	Ignoring	Sensitive communication		
P12	Transparent management	Psychological support and guidance	Initiating an investigation	Initiating an investigation

## Data Availability

Data will be made available on request.
